# A new technique for closure of pericardial defects: pericardial rug weave

**DOI:** 10.1186/s12893-024-02368-5

**Published:** 2024-04-13

**Authors:** Göktürk Fındık, Mehmet Çetin, Hakan Nomenoğlu, İlteriş Türk, Serdar Acemoğlu, Necati Solak, Mehmet Ali Can

**Affiliations:** 1 Department of Thoracic Surgery, Ataturk Sanatoryum Training and Research Hospital, Ankara, Turkey; 2 Department of Thoracic Surgery, Omer Halisdemir University Training and Research Hospital, Niğde, Turkey; 3 Department of Thoracic Surgery, Sincan State Hospital, Ankara, Turkey; 4 Department of Radiology, Ataturk Sanatoryum Training and Research Hospital, Ankara, Turkey

**Keywords:** Complication, Herniation, Intrapericardial, Pericardial mesh, Pneumonectomy

## Abstract

**Background:**

Pericardial defect that occurs after intrapericardial pneumonectomy can cause many fatal complications, and closing the defect with mesh is a widely used surgical method to prevent these complications.

**Methods:**

Data of patients who underwent intrapericardial pneumonectomy and pericardial resection in our clinic between October 2010 and June 2022 were retrospectively reviewed. Patients were divided into two groups, those who had prolene mesh used to close the pericardial defect and those who underwent the “Rug Weave” technique we proposed as an alternative, and the results were compared.

**Results:**

The study included 23 patients, one of whom was female. All patients underwent surgery due to malignancy. The vast majority of the patients had a diagnosis of squamous cell lung carcinoma (86.9%). Atrium was added to three patients and rib resection was added to one patient during intrapericardial pneumonectomy and pericardial resection. There was no significant difference between the two groups in terms of average age, gender, and length of hospital stay. There was no significant difference between the two groups in terms of complications, including atrial fibrillation, which is commonly seen in these patients (*p* = 0.795). The Rug Weave group had an average defect width of 23.96 cm2 and was found to be advantageous in terms of overall survival compared to the mesh group (*p* = 0.017).

**Conclusions:**

The “Rug Weave” technique we proposed for closing pericardial defects after pneumonectomy can be used as a cheaper method safely and effectively that reduces complications as much as the traditional method of using mesh.

## Background

Acute postoperative cardiac herniation is an extremely rare but fatal complication of intrapericardial pneumonectomy. Diagnostic clues and rapid revision are emphasized in the current literature, which are important for the management of this serious complication. In this situation, which has a mortality of approximately 50% even with rapid intervention, intraoperative measures are of great importance to prevent herniation [1]. Direct suturing of the pericardium or closure with a patch are among them. These methods may cause complications such as rupture of the sutures due to tissue tension and weakness, opening of the defect, infection due to the patch, arrhythmia, tamponade, and constrictive pericarditis [2].

In the present study, we share our recommendations for the closure of defects formed after intrapericardial pneumonectomy with a less complicated, different, and useful method, which we call pericardial rug weave.

## Methods

The study included patients who underwent intrapericardial pneumonectomy with pericardial resection performed at our clinic between October 2010 and June 2022. The following data were collected retrospectively: age, sex, type of resection performed, operative side, postoperative pathology, tumor size, invasion status of the mass, pericardial defect width, technique used for defect closure, pathological tumor stage, postoperative complications, length of hospital stay, and survival data of the patients.

Ethics committee approval numbered 2012-KAEK-15/2583 was obtained from Ankara Atatürk Sanatorium Training and Research Hospital Clinical Research Ethics Committee before the study. The study was prepared in accordance with the Declaration of Helsinki.

### Surgical technique

**Fig. 1:**
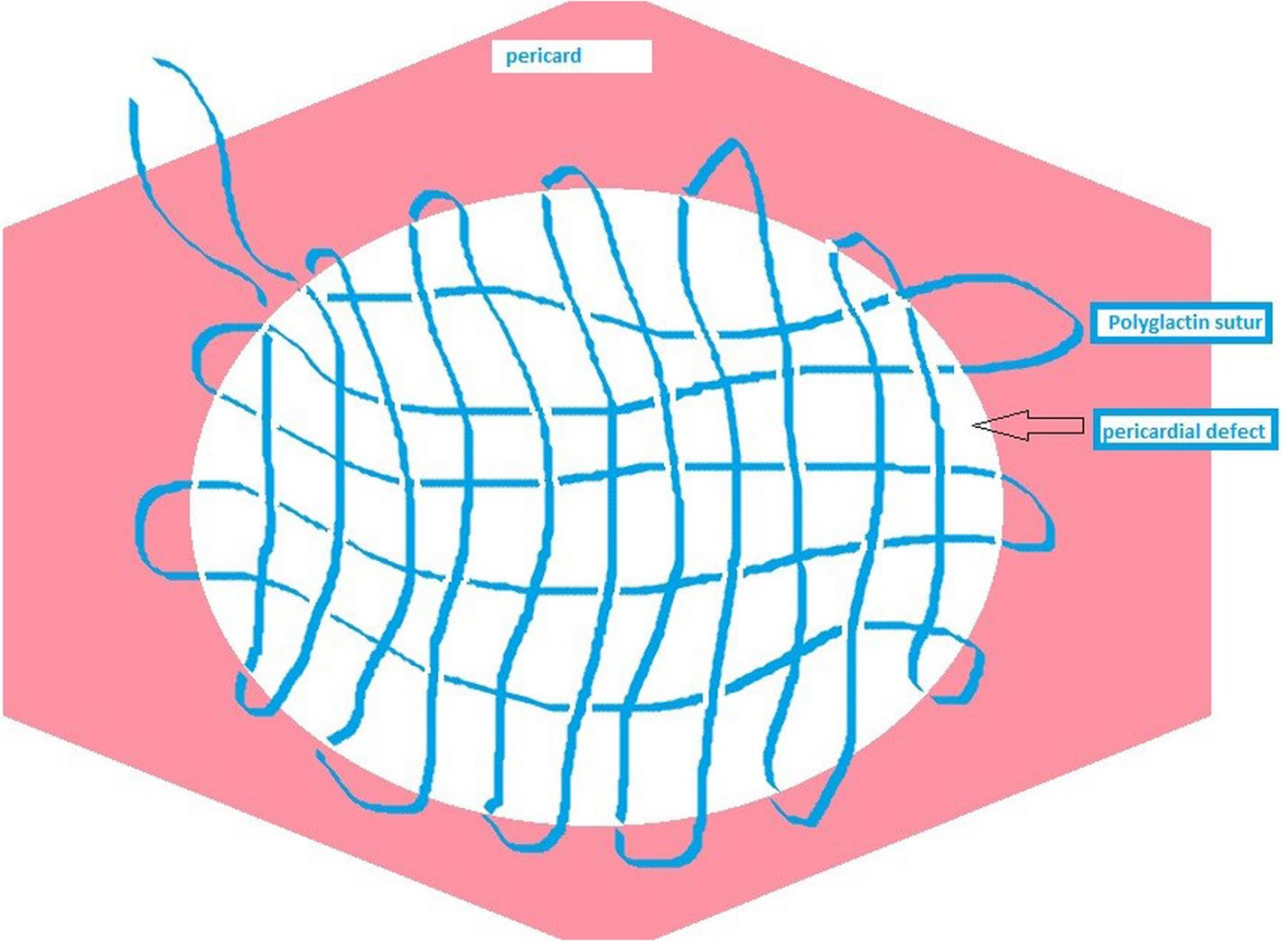
Procedure for the pericardial rug weave and aschematic drawing of it

**Fig. 2:**
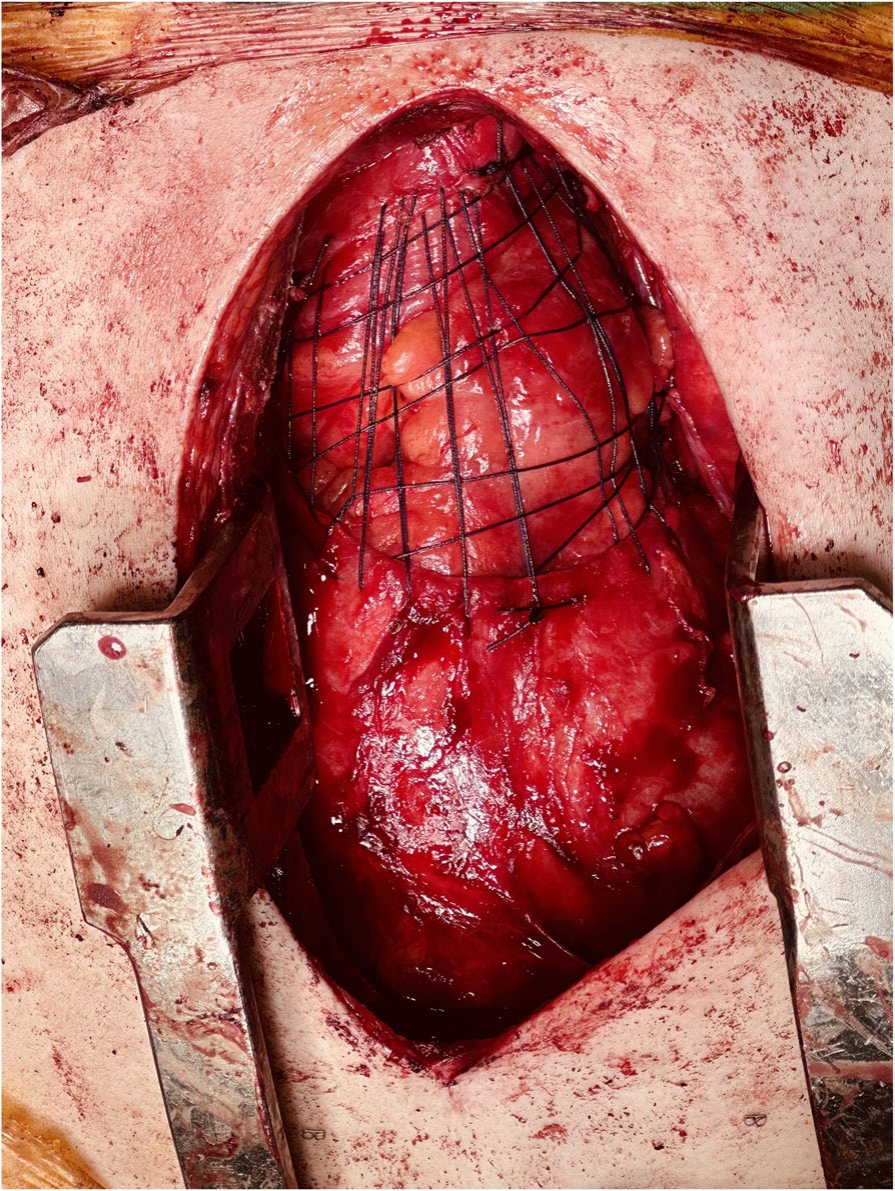
Intraoperative view of the pericardial rug weave

The patients were divided into two groups. In the “mesh group”, the defect was repaired using prolen mesh, while in the “Rug Weave group”, polyfilament 2 − 0 and 3 − 0 polyglactin sutures were used to repair the pericardial defect after resection. To prevent pericardial cardiac herniation and provide drainage, the pericardium was stitched from one outside to the other. An intraoperative photograph of the technique illustrated in Fig. 1 is provided in Fig. 2. We call this technique as “Fındık’s technique”.

### Statistical analysis

The statistical analyses of the study were evaluated using the SPSS 24.0 software package. Descriptive statistics were presented as the number of units (n), percentage (%), mean ± standard deviation (mean ± sd) for age, and median (min - max) for follow-up and operative times. When the surgical technique was grouped as pericardial rug weave and mesh repair, continuous numerical variables between the two groups were compared using Mann-Whitney U test. The distribution of categorical variables between the groups was evaluated using the Pearson’s chi-square analysis and Fisher’s Exact test. A *p*-value of less than 0.05 was considered statistically significant.

## Results

Between October 2010 and June 2022, a total of 23 patients who underwent intrapericardial pneumonectomy with pericardial resection were included in the study. Patient data including age, sex, tumor size, invasion, operation year, diagnosis, type of surgery, pathological tumor stage, complications, and length of hospital stay are shown in Table 1. All patients underwent surgery due to malignancy, with 15 patients (65.2%) undergoing pneumonectomy, 4 patients (17.4%) undergoing pneumonectomy after neoadjuvant chemotherapy, 3 patients (13%) undergoing pneumonectomy with atrium resection, 16 cases of left and 7 cases of right pneumonectomy were observed. 1 patient (4.3%) undergoing complementary extended pneumonectomy and rib resection. SCC was diagnosed in 20 patients (86,9%), large cell lung carcinoma in 2 patients (8.7%) and combined small and large cell carcinoma in 1 patient (4.3%). The diameter of the pericardial defect was not available for all patients who underwent mesh repair except for one patient, as the data was too old. In the mesh group, the defect size was reached in only one patient and was measured as 27.09 cm^2^. However, the median defect size in 12 patients who underwent peridarchial rug weave was 23.43 cm^2^ (min 17.31 - max 32.73).

**Table 1:** General characteristics of the patients, operational information, postoperative pathology, and survival-complication results

Case	Age	Sex	Side	Tumor size (cm)	Year of Surgery	Operation	Local invasion	Diagnosis	Technic	Stage	Defect Size (cm^2)^	Complication	Length of Stay
**1**	66	Male	Left	7	2018	IP	Parietal Pericardium	SCC	Rug Weave	T4N1M0	32,73	BPF	14
**2**	65	Male	Left	6	2022	Complement Ip + 6-7-8. Ribs Resection	Parietal Pericardium, Ribs	LCLC	Rug Weave	T3N0M0	23,81	None	7
**3**	59	Male	Right	6.7	2022	IP + Atrium Resection	Atrium	SCC	Rug Weave	T4N1M0	23,04	Atrial Fibrillation	13
**4**	63	Male	Left	5.1	2022	IP	Parietal Pericardium	SCC	Rug Weave	T3N1M0	28,36	None	6
**5**	64	Male	Left	5	2022	IP	Parietal Pericardium	SCC	Rug Weave	T4N1M0	17,31	None	8
**6**	61	Female	Left	7.2	2022	Neoadjuvant chemotherapy + IP	Parietal Pericardium	SCC	Rug Weave	T4N2M0	19,98	None	7
**7**	75	Male	Left	4.7	2021	IP	Parietal Pericardium	SCC	Rug Weave	T3N0M0	27,99	None	7
**8**	63	Male	Left	3.8	2021	IP	Parietal Pericardium	SCC	Rug Weave	T3N1M0	22,79	None	7
**9**	63	Male	Left	4.2	2022	Neoadjuvant chemotherapy + IP	Parietal Pericardium	SCC	Rug Weave	T3N1M0	27,52	Pneumonia + PTE	15
**10**	72	Male	Left	5.3	2019	IP	Parietal Pericardium	SCC	Rug Weave	T3N1M0	19,03	Atrial Fibrillation	7
**11**	56	Male	Left	8	2021	IP	Parietal Pericardium	SCLC + LCLC	Rug Weave	T4N0M0	24,41	Atrial Fibrillation	7
**12**	50	Male	Right	3	2019	IP	Parietal Pericardium	SCC	Rug Weave	T3N2M0	20,57	None	6
**13**	47	Male	Left	7	2018	IP	Parietal Pericardium	SCC	Mesh	T4N2M0	27,09	None	14
**14**	53	Male	Right	4	2012	IP	Parietal Pericardium	SCC	Mesh	T4N2M0	-	None	9
**15**	51	Male	Right	20	2012	Neoadjuvant chemotherapy + IP	Parietal Pericardium	LCLC	Mesh	T4N0M0	-	None	8
**16**	68	Male	Right	12	2011	IP + Atrium Resection	Atrium	SCC	Mesh	T4N0M0	-	Atrial Fibrillation	6
**17**	54	Male	Right	4	2011	Neoadjuvant chemotherapy + IP	Parietal Pericardium	SCC	Mesh	T3N1M0	-	BPF + Pneumonia	8
**18**	77	Male	Left	4.2	2011	IP	Parietal Pericardium	SCC	Mesh	T4N2M0	-	Atrial Fibrillation + BPF + Pneumonia	21
**19**	58	Male	Left	10	2011	IP + Atrium Resection	Atrium	SCC	Mesh	T4N1M0	-	None	17
**20**	58	Male	Left	5	2011	IP	Parietal Pericardium	SCC	Mesh	T3N2M0	-	None	9
**21**	45	Male	Left	6	2010	IP	Parietal Pericardium	SCC	Mesh	T3N1M0	-	None	6
**22**	63	Male	Left	6	2010	IP	Parietal Pericardium	SCC	Mesh	T3N1M0	-	None	11
**23**	48	Male	Right	3	2010	IP	Parietal Pericardium	SCC	Mesh	T3N1M0	-	Atrial Fibrillation	8

*SCC *Squamous Cell Carcinoma, *SCLC *Small Cell Lung Carcinoma, *LCLC *Large Cell Lung Carcinoma, *IP *Intrapericardial Pneumonectomy, *BPF *Bronchopleural fistula

When the operative times are considered, the median operative time of patients who underwent pericardial rug weave is 215 (min 125 - max 265) minutes. The operative time for only one patient in the mesh group was reached and was found to be 280 minutes.

 When comparing the mean age, gender, stage, complications, hospitalization duration, and defect size between the pericardial rug weaving and mesh repair techniques, no statistically significant difference was observed (*p* = 0.071, 1.00, 0.478, 1.00, 0.151, 0.593). The distribution of patients is shown in Table 2.

**Table 2:** Statistical comparison of demographic and disease data of the patients

	Rug Weave (*n* = 12)	Mesh (*n* = 11)	Total (*n* = 23)	p	
Mean Age	63,08 ± 6,58	56,55 ± 9,71	59,96 ± 8,7	0,071	*t* = 1,91
Gender				1,00	_*x*_^*2*^= 0,958
Male	11 (%91,7)	11 (%100)	22 (%95,7)		
Female	1 (%8,3)	0 (%0)	1 (%4,3)		
Stage				0,478	_*x*_^*2*^= 2,008
II	2 (%16,7)	0 (%0)	2 (%8,7)		
III	10 (%83,3)	11 (%100)	21 (%91,3)		
Complication				1,00	_*x*_^*2*^= 0,062
Exists	5 (%41,7)	4 (%36,4)	9 (%39,1)		
Doesn’t exist	7 (%58,3)	7 (%63,6)	14 (%60,9)		
Length of Stay (median (min,max))	7 (min = 6, max = 15)	9 (min = 6, max = 21)	8 (min = 6, max = 21)	0,151	*z*= -1,436

When evaluating the complications, 14 patients (60.9%) did not experience any complications. One patient (4.3%) had only bronchopleural fistula (BPF), 5 patients (21.7%) had only atrial fibrillation (AF), 1 patient (4.3%) had both pulmonary thromboembolism (PTE) and pneumonia, 1 patient (4.3%) had both BPF and pneumonia, and 1 patient (4.3%) had AF, BPF, and pneumonia. Atrial fibrillation was observed in 3 patients in each group. There was no significant difference between the two groups in terms of the incidence of complications (*p* = 1,00).

Considering the follow-up periods, only one of our patients who underwent pericardial rug weave deceased in the third postoperative month. The median follow-up period of patients who underwent pericardial rug weave is 15.5 months (min 3 – max 53). The mean survival time of patients who underwent pericardial mesh repair is 25.25 ± 9.98 months.

## Discussion

Postoperative cardiac herniation, which was first reported by Bettman in 1948, is still among the complications causing the highest mortality in patients who have undergone pericardial resection [3]. It is an issue that should be considered during the entire perioperative period, especially in cases of late diagnosis, due to its nearly 100% mortality [1].

Since it is quite rare, acute complications are mentioned in the literature in case reports, despite the pericardial defect having been repaired. Especially in the postoperative period, findings such as sudden deterioration of general condition, superior vena cava syndrome, hypotension, and ventricular fibrillation are important indicators of acute cardiac herniation. Revision surgery should be performed after the patient is positioned for lateral decubitus on the non-operated side, and the heart should be placed in its natural position without strangulation. When strangulation develops, the mortality rate reaches 100% [4–6]. There is also a case report stating that in cases of herniation, the administration of 2000 cc of saline solution to the pneumonectomy pouch by placing the patient’s lateral decubitus on the opposite side of the operation also corrects the herniation [7]. Despite all this, even with early intervention there is 50% mortality rate. Therefore, intraoperative repair of the defect is of great importance [1].

During the intraoperative period, repair of the defect, even if it is small, is recommended. There are different techniques for repair, such as direct suturing of the pericardium or repair with a patch [8, 9]. In direct pericardial suturing, if the tear and defect area continues to expand due to tissue tension, and in cases where pericardial fluid drainage cannot be achieved when a patch has been used, tamponade and direct patch-related constrictive complications such as pericarditis can occur. In order to minimize these risks, polytetrafluoroethylene (PTFE) is often used as a patch, but PTFE also has risks, albeit less [10, 11]. In our study, we did not observe a significant difference in terms of complications between the mesh we used before applying the pericardial rug weave technique. However, to avoid the potential complications of the mesh, we started using the newly defined technique in our surgeries since 2018. The advantage of this method is that it does not create pericardial tension, especially preventing tears, and at the same time there is almost no risk of infection and tamponade.

Intrapericardial pneumonectomy is primarily utilized for patients with locally advanced lung cancer [2, 12]. Furthermore, extrapleural pneumonectomy with pericardial resection is performed during surgical procedures for mesothelioma. In such cases, the pericardium is entirely removed, leaving a defect too extensive to be repaired through suturing [10]. Thus, our described technique can primarily be utilized in cases of lung malignancies, and occasionally for smaller defects such as in non-malignant intrapericardial pneumonectomy performed for bronchiectasis. The proximity of the working area of lung surgery to the atrium due to veins poses a risk for auricles to herniate from even minor defects, which may result in continuous irritation through friction on the pericardial leaf’s edge. This can potentially initiate a process leading to complications such as embolism and arrhythmia [10]. However, in our study, we did not observe a significant difference in cardiac complications either. Although the defect size was only detected in one patient in the mesh group, 23.43 cm^2^ median value was observed in the rug weave group. It can be safely used at these levels. Although there are no significant results in the literature regarding the repaired defect diameter, except for a case of herniation that developed from a 4 × 4 cm^2^ defect in a case presentation, the relationship between size and success is open to evaluation with new studies [13].

Despite no statistically significant differences observed in patient demographics such as age, gender, stage our study demonstrates that pericardial rug weave technique can be safely used with similar efficacy as mesh repair, albeit with a lower number of cases. However, despite there being no difference in pathological stages, we believe that survival rates being significantly better in the rug weave group should not be the only factor taken into account when assessing the effectiveness of the technique.

The value of the present study is that this new technique developed to counter morbidity and mortal complications in acute cardiac herniation, which is one of the most serious complications of intrapleural pneumonectomy, does not include the complications of other repair methods. In cases of empyema that will develop in the pneumonectomy pouch, the mesh should be eliminated, but since there is no mesh in our newly defined technique, new surgery will not even be necessary. Of course, with an increase in the number of patients, the contribution of the technique to practice will be seen more clearly.

## Conclusions

In conclusion, pericardial rug weaving is a safe and effective method for closing pericardial defects after intrapericardial pneumonectomy. Pending further studies, the rug weave method could be regarded as the top option, given that the risks of complications related to the defect and its repair do not differ significantly.

## Data Availability

The datasets used and/or analysed during the current study are available from the corresponding author on reasonable request.
